# DNA Barcoding for Identification of ‘*Candidatus* Phytoplasmas’ Using a Fragment of the Elongation Factor Tu Gene

**DOI:** 10.1371/journal.pone.0052092

**Published:** 2012-12-18

**Authors:** Olga Makarova, Nicoletta Contaldo, Samanta Paltrinieri, Geofrey Kawube, Assunta Bertaccini, Mogens Nicolaisen

**Affiliations:** 1 Department of Agroecology, AU Science and Technology, Aarhus University, Slagelse, Denmark; 2 Alma Mater Studiorum, University of Bologna, DipSA – Plant Pathology, Bologna, Italy; The University of Hong Kong, China

## Abstract

**Background:**

Phytoplasmas are bacterial phytopathogens responsible for significant losses in agricultural production worldwide. Several molecular markers are available for identification of groups or strains of phytoplasmas. However, they often cannot be used for identification of phytoplasmas from different groups simultaneously or are too long for routine diagnostics. DNA barcoding recently emerged as a convenient tool for species identification. Here, the development of a universal DNA barcode based on the elongation factor Tu (*tuf*) gene for phytoplasma identification is reported.

**Methodology/Principal Findings:**

We designed a new set of primers and amplified a 420–444 bp fragment of *tuf* from all 91 phytoplasmas strains tested (16S rRNA groups -I through -VII, -IX through -XII, -XV, and -XX). Comparison of NJ trees constructed from the *tuf* barcode and a 1.2 kbp fragment of the 16S ribosomal gene revealed that the *tuf* tree is highly congruent with the 16S rRNA tree and had higher inter- and intra- group sequence divergence. Mean K2P inter−/intra- group divergences of the *tuf* barcode did not overlap and had approximately one order of magnitude difference for most groups, suggesting the presence of a DNA barcoding gap. The use of the *tuf* barcode allowed separation of main ribosomal groups and most of their subgroups. Phytoplasma *tuf* barcodes were deposited in the NCBI GenBank and Q-bank databases.

**Conclusions/Significance:**

This study demonstrates that DNA barcoding principles can be applied for identification of phytoplasmas. Our findings suggest that the *tuf* barcode performs as well or better than a 1.2 kbp fragment of the 16S rRNA gene and thus provides an easy procedure for phytoplasma identification. The obtained sequences were used to create a publicly available reference database that can be used by plant health services and researchers for online phytoplasma identification.

## Introduction

Phytoplasmas are bacterial plant pathogens that are transmitted by hemipteran insect vectors and that cause significant losses in agricultural production worldwide [1]. They are assigned to a clade within the class *Mollicutes,* a branch of the Gram-positive eubacteria that lack cell walls [2]. Although phytoplasmas are relatively well studied, their identification is still challenging as they do not possess a distinctive morphology and are currently non-culturable *in vitro*.

Phytoplasmas infect over 200 plant species [1], and, when infected, plants show symptoms such as virescence, phyllody, yellowing, witches’ broom, and generalized decline. Apple proliferation, ‘stolbur’, ‘flavescence dorée’, ‘bois noir’ and coconut lethal yellowing are among the most prominent phytoplasma diseases and are considered of quarantine relevance in the EU, which means that their spread should be especially tightly regulated. No fully resistant crop varieties are currently available, and main disease management strategies are limited to control of insect vectors (when known), and elimination of infected/symptomatic plants. Therefore, availability of reliable and efficient methods for identification is especially important for this group of pathogens.

Currently, over thirty species are recognized within the ‘*Candidatus* Phytoplasma’, mostly based on at least 97.5% sequence identity within their 16S ribosomal RNA gene, but also on biological, phytopathological, and other molecular characteristics [3]. For practical diagnostics purposes, phytoplasmas are commonly classified based on patterns of restriction fragment length polymorphism (RFLP) analysis of a 1.2 kbp fragment of the 16S rRNA gene [4], [5] after amplification with R16F2n and R16R2 [6] primers, often preceded by nested PCR amplification of a 1.8 kb fragment using P1 [7] and P7 [8] primers. However the 16S rRNA gene does not show much variation and may be present in two, sometimes non-identical, copies [9]. This has prompted the use of other, more variable regions of the phytoplasma genome. The 16SrI (‘*Ca.* P. asteris’-related) group has been subdivided using the *tuf* gene, the ribosomal protein operon (*rp*) and the 16S–23S rRNA intergenic spacer region [10], [11], along with the *secY* gene [12] and *groEl* gene [13]. The 16SrV group has been subdivided using *secY*, *map* and *uvrB–degV* [14], and *rp* [15] genes. The 16SrXII group has been subdivided using the *tuf* gene and the *rp* operon [16].The majority of these studies have examined taxonomic relations within specific 16Sr groups, as these PCR primers are group-specific and do not amplify DNA from phytoplasmas in other groups. Martini et al. (2007) used primers that amplify a 1,2–1,4 kbp fragment of the genes *rplV* (*rpl22*) and *rpsC* (*rps3*) from a wide range of phytoplasmas and constructed a phylogenetic tree which resulted in a finer resolution within 16S ribosomal groups [17]. Lee and coworkers (2010) used various primers for different phytoplasma groups that amplified a fragment of more than 2 kb of the *secY* gene and were also able to construct a phylogenetic tree with high resolution [18]. Although these studies improved knowledge on phylogenetic relationships, the long size of amplicons makes them rather impractical for routine sequencing-based identification. The first attempt to use a shorter marker for universal phytoplasma identification was undertaken by Hodgetts *et al*. (2008), when a 480 bp-long fragment of the *secA* gene was used [19]. The resulting phylogenetic tree revealed a good resolution of ‘*Candidatus* Phytoplasma’ species and the *secA* fragment emerged as a promising marker for universal identification of phytoplasmas. However, under-representation of phytoplasma strains (34 strains in total) in the study did not allow evaluation of its full potential, and furthermore, some strains, which were not tested in the original study, did not amplify well using the published primers, at least in our hands.

It has been suggested that universally amplified, short, and highly variable DNA markers (DNA barcodes) may help to rapidly identify organisms to a species level with a high confidence in a cost-effective way, which would be useful in a wide array of applications, including diagnostics [20], [21]. In general, DNA barcodes should contain sufficient variation to discriminate among closely related species and yet possess highly conserved regions so that the barcode region can be easily amplified and sequenced with standard protocols. Furthermore, the taxonomy to which DNA barcodes are linked should be already established by other means, as DNA barcoding is considered a method of molecular identification and not taxonomy [22]. Indeed, DNA barcoding recently emerged as a popular and convenient tool for species identification and has been successfully used and taxonomically validated for eukaryotes [23]–[25]. Several international DNA barcoding projects have been launched in recent years [26]. QBOL (Quarantine Barcode of Life), an EU project aiming at the development of a universal identification system for main groups of quarantine organisms, including phytoplasmas, was started in 2009 [27]. As a part of this initiative we attempted to develop a universal DNA barcoding-based tool for phytoplasma identification.

Here, we present the results of a study on DNA barcoding for phytoplasma identification. A set of primers amplifying a fragment of the *tuf* gene was designed and the potential of this fragment as a DNA barcode was examined. Elongation factor Tu is a key protein involved in the process of translation that is present in all known organisms, relatively well conserved and found as a single copy in the four phytoplasma genomes fully sequenced to date [28]–[31]. Successful amplification and sequencing of the 91 phytoplasma strains tested, and ability to separate various phytoplasma strains to ‘*Candidatus*’ species, 16S rRNA group and subgroup levels suggested it can be used as a DNA barcode for phytoplasma identification.

## Materials and Methods

### Taxon Selection and Nucleic Acid Preparation

**Figure 1:**
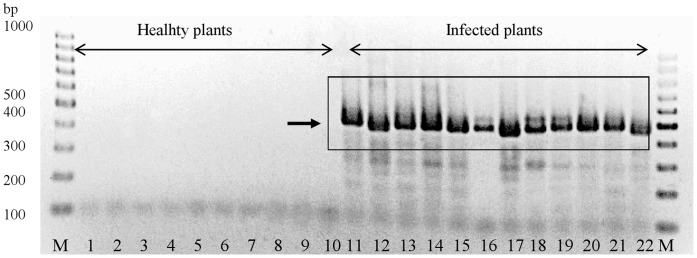
PCR amplification of the 420–440 **bp** ***tuf*** **barcode from phytoplasma-infected plants and absence of amplification from healthy plants.** Lanes 1–10: healthy plants; lanes 11–22: phytoplasma-infected plants. Lanes: 1–apple, 2–aster, 3– grapevine, 4–lettuce, 5–maize, 6–tobacco, 7–periwinkle, 8–plum, 9–potato, 10–oat, 11–CA, 12–CoP, 13–FD-AS, 14–JR-1, 15–LUM, 16–NJ-AY, 17–PrB, 18–RuS, 19–AP-15, 20–ASHY4, 21–ASLO, 22–BF, lanes M–1 kb DNA ladder.

Ninety one phytoplasma strains collected in various geographic locations from 16Sr groups -I, -II, -III, -IV, -V, -VI, -VII, -IX, -X, -XI, -XII, -XV, -XX were used in this study. The strain names and their respective ‘*Candidatus* Phytoplasma’ species (when available), 16Sr group and subgroup, geographical origin and host plant are listed in **Table S1**. The majority of the strains were obtained from the phytoplasma reference collection located at the University of Bologna, Italy [32], or as DNA preparations from other researchers or were maintained in periwinkle (*Catharanthus roseus*), or in napier grass (*Pennisetum purpureum*) for napier grass stunt, in grapevine for ‘flavescence dorée’ and ‘bois noir’ strains. Healthy apple, aster, grapevine, lettuce, maize, tobacco, periwinkle, plum, potato and oat plants, some of which are typical hosts of phytoplasmas, were used for negative controls (**Figure 1**). DNA was extracted by the method described in Prince *et al*. 1993 [33].

### Primer Design

*tuf* gene sequences of phytoplasmas (16Sr groups -I, -III, -IV, -V, -VII, -X and -XII), several plant species, *Bacillus* and *Clostridium* spp. were retrieved from the NCBI GenBank (http://www.ncbi.nlm.nkh.gov/), aligned using the DNA Workbench (CLCbio, Aarhus, Denmark) software using default settings, and conserved regions present in all phytoplasmas were identified. Primer sequences were designed by visual assessment of the alignment to include all phytoplasma groups and to exclude plant and bacterial DNA.

### PCR Amplification and Sequencing

PCR was carried out in a 25 µl reaction mixture containing 10 µM primers, Fermentas *Taq* polymerase (for the P1/P7 primer pair) or Promega GoTaq DNA polymerase (for all other primers) and respective reaction buffers, 25 mM MgCl_2_, 10 mM dNTPs mix (Fermentas) and sterile water. One µl (20 ng/µl) of DNA template was used per reaction. For amplification of the *tuf* barcode, two pairs of primer cocktails (Tuf340/Tuf890 for direct PCR and Tuf400/Tuf835 for nested PCR, **Table 1**) were used. Each primer cocktail consisted of several variants of the same primer mixed in equimolar proportions to the final concentration of 10 µM. The same primer cocktails were used for amplification of all phytoplasma strains used in this study. PCR conditions for both rounds of PCR with primer pair Tuf340/Tuf890 followed by primer pair Tuf400/Tuf835 (**Table 1**) were 94°C for 3 min followed by 35 cycles of 94°C for 15 sec, 54°C for 30 sec and 72°C for 1 min and a final extension step of 72°C for 7 min. Resulting PCR products from the first round were diluted 1∶30 with sterile water and 1 µl product was used in the nested PCR assay. Amplification of the 16S rRNA gene was performed in a nested PCR assay using primers P1 and P7 in the first round, followed by primer pairs P1-ATT (AAGAGTTTGATCCTGGCTCAGG)/P625 (ACTTAYTAAACCGCCTACRCACC) (this study), P4 [8]/P7, 16R758f (M1) [34]/P7 and R16F2n/R16R2 [35] in the nested PCR to allow for overlapping coverage of the 1,800 bp region. The cycling conditions for the primer pair P1-ATT/P625 were 94°C for 3 min followed by 35 cycles of 94°C for 15 sec, 64°C for 30 sec and 72°C for 1 min and a final extension step 72°C for 7 min; for the primer pair P4/P7 94°C 3 min, 35 cycles of 94°C for 15 sec, 52°C for 30 sec, 72°C for 60 sec followed by final extension 72°C for 7 min; for the primer pair M1/P7 94°C for 5 min, followed by 35 cycles of 94°C for 90 sec, 54°C for 2 min, 72°C for 3 min, followed by 72°C for 7 min; for the primer pair R16F2n/R16R2 94°C for 2 min, followed by 35 cycles of 94°C for 1 min, 50°C for 2 min, 72°C for 3 min, followed by 72°C for 10 min. PCR products from the first round with P1/P7 primers were diluted 1∶30 with sterile water and 1 µl product was used in the nested PCR assays. Post-PCR cleanup and sequencing of the amplicons were processed by Macrogen Inc. (Seoul, Korea). All PCR products were sequenced on both strands using M13F and T7 as primers for *tuf* sequences and P1, P625, P4, P7, Phyt16Sr (TCCTACGGGAGGCAGCAG), M1, 16R1232r(M2) [34] and Phyt16Sr2 (TATTGTTAGTTGCCAGCACG) for the 16Sr gene.

**Table 1:** Primers used for amplification of the *tuf* DNA barcode.

Primer cocktail	Position in AYWB *tuf* gene	Primer cocktail components	Primer sequence	Proportionsof each primerin a primercocktail	Notes
Tuf340	157–179	Tuf340a	GCTCCTGAAGAAARAGAACGTGG	1∶1	used as aforward
		Tuf340b	ACTAAAGAAGAAAAAGAACGTGG		primer mixin a directPCR assay
Tuf400	211–236	Tuf400aM13F	GTAAAACGACGGCCAGTGAAACAGAAAAACGTCAYTATGCTCA		
		Tuf400bM13F	GTAAAACGACGGCCAGT GAAACTTCTAAAAGACATTACGCTCA		used as aforward
		Tuf400cM13F	GTAAAACGACGGCCAGTGAAACATCAAAAAGACAYTATGCTCA	1∶1:1∶1:1	primer mix ina nested
		Tuf400dM13F	GTAAAACGACGGCCAGTGAAACAGAAAAAAGACAYTATGCTCA		PCR assay
		Tuf400eM13F	GTAAAACGACGGCCAGTCAAACAGCTAAAAGACATTATYCTCA		
Tuf835	628–654	Tuf835raT7	TAATACGACTCACTATAGGGAACATCTTCWACHGGCATTAAGAAAGG		used as a reverse
		Tuf835rbT7	TAATACGACTCACTATAGGGAACACCTTCAATAGGCATTAAAAAWGG	1∶1:1	primer mix in a nested
		Tuf835rcT7	TAATACGACTCACTATAGGG AACATCTTCTATAGGTAATAAAAAAGG		PCR assay
Tuf890	685–710	Tuf890ra	ACTTGDCCTCTTTCKACTCTACCAGT		used as a reverse
		Tuf890rb	ATTTGTCCTCTTTCWACACGTCCTGT	1∶1:1	primer mix in a direct PCR assay
		Tuf890rc	ACCATTCCTCTTTCAACACGTCCAGT		

Two pairs of primer cocktails were used for universal amplification of the *tuf* barcode from all phytoplasma strains employed in this study in a nested PCR assay. Tuf 340/Tuf890 and Tuf400/Tuf835 primer cocktails were used in direct and nested PCR respectively. Each primer cocktail contained slightly different variants of the same primer mixed in equimolar amounts. The nucleotide sequences of the general sequencing primers M13F and T7 are underlined with a single and a double line, respectively. Primer positions correspond to the positions in the *tuf* gene of ‘*Ca.* P. asteris’ strain AY-WB (Genbank accession number CP000061).

### Sequence and Phylogenetic Analyses

**Figure 2:**
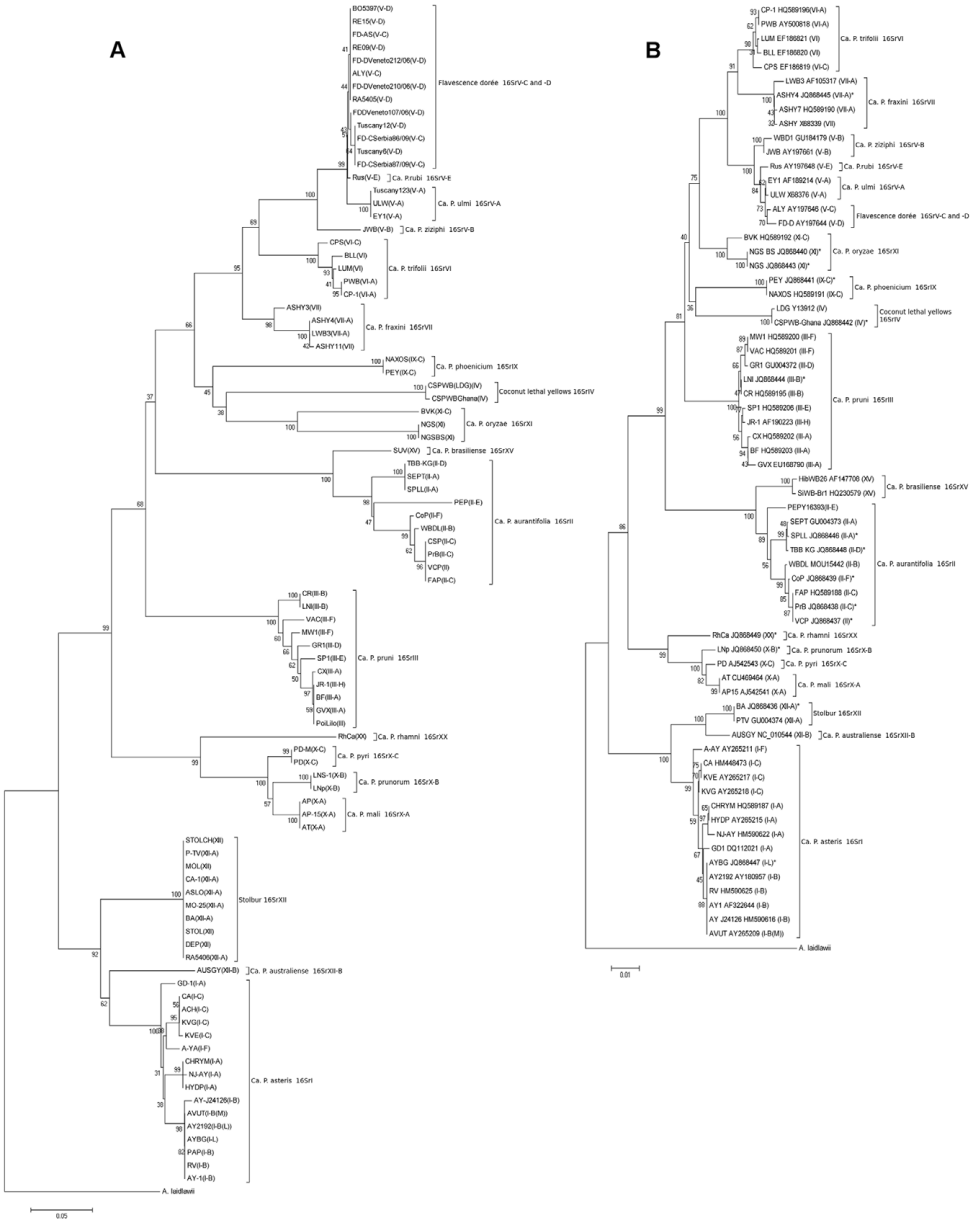
NJ trees of the *tuf* barcode (a) and the R16F2n/R16R2 fragment of the 16S ribosomal RNA gene (b). The *tuf* barcode tree largely follows the branching pattern of the 16S rRNA tree. Numbers at the nodes indicate bootstrap values; bars, substitutions per nucleotide position; asterisk, strains, whose 16S rRNA gene was sequenced in this study; 16S rRNA GenBank sequence accession number is indicated following the strain acronym; 16Sr group and subgroup are in parentheses; *A. laidlawii* (accession number NC010163) was used as an outgroup.

Sequences were assembled and edited using DNA Workbench (CLC bio, Aarhus, Denmark) software. The resulting consensus sequences have been deposited in the NCBI GenBank and the Q-Bank (http://www.q-bank.eu/) databases. The NCBI GenBank accession numbers of the *tuf* barcode sequences can be found in **Table S1.** The NCBI GenBank accession numbers of the 16S rRNA gene sequences sequenced in this study or obtained from the NCBI GenBank for phylogenetic analyses can be found in **Figure 2** **.** The *tuf* gene sequence of *Acholeplasma laidlawii* (accession number NC010163) was retrieved from the NCBI GenBank**.** The 1.2 kb R16F2n/R16R2 fragment of the 16S rRNA gene from 66 phytoplasma strains and the 420–444 bp Tuf400/Tuf835 fragment of the *tuf* gene from 91 strains were used for construction of the 16S rRNA and *tuf* alignments, respectively. Sequence alignments were performed using a progressive alignment algorithm [36] implemented in the DNA Workbench package (CLC bio, Aarhus, Denmark) with the following settings: gap open cost 10, gap extension cost 1, end gap cost as any other. The alignments were exported to the MEGA 4 software [37] for distance and phylogenetic analyses. Neighbor-Joining (NJ) [38] trees were constructed using 500 replicates for bootstrap analysis [39] and *A. laidlawii* as an outgroup to root the tree. Average intra- and inter-group evolutionary divergences were calculated using the Kimura 2-parameter (K2P) distance model [40]. Groups for determining genetic distances were defined based on the 16Sr [4], [5] and ‘*Ca.* Phytoplasma’ (when applicable) [3] classification systems. The bootstrap [39] consensus Maximum likelihood (ML) trees were inferred from 100 replicates using the best fitting model of 24 different nucleotide substitution models (**Table S2 and Table S3**). The Tamura-Nei model [41] was used to infer the 16S rRNA ML tree, and the Tamura 3-parameter model [42] was used for construction of the *tuf* ML tree. In both cases a discrete Gamma distribution (+G) was used to model evolutionary rate differences among sites, assuming that a fraction of sites are evolutionarily invariable (+I).

## Results and Discussion

For an ideal phytoplasma DNA barcoding procedure, the barcode should be easily amplifiable using a single set of primers, be relatively short to facilitate sequencing, show non-overlapping inter- and intra-species sequence divergence (i.e. creating the DNA barcoding gap, when interspecific variation is normally greater than intraspecific variation by an order of magnitude) [22] and it should provide sufficient resolution for identification of phytoplasma ‘*Candidatus*’ species within the current taxonomy. Moreover, DNA barcoding of uncultivable plant pathogenic bacteria obviously requires that primers do not amplify plant or unrelated bacterial DNA. A DNA barcoding-based system was developed in this study that fulfilled these criteria and allowed identification of all tested phytoplasma strains to ribosomal group and/or phytoplasma ‘*Candidatus*’ species level and in some cases to subgroup level with only one set of nested primers.

### *Tuf* Barcode Primer Design, Amplification and Sequencing

Prior to this study, *tuf* gene sequences available in the NCBI nucleotide database were limited to a few sequences from the 16Sr groups -I, -III, -IV, -V, -VII, -VIII, -X and -XII. Alignment of these phytoplasma *tuf* sequences resulted in identification of conserved regions within the *tuf* gene. These regions were exploited for primer design in an attempt to amplify *tuf* gene sequences from most or all phytoplasmas, but not from plant or DNA from other bacteria. As phytoplasmas can occur in low titer, two sets of primers were developed for use in a nested PCR assay, resulting in four primer cocktails to accommodate any sequence variation between phytoplasma groups: Tuf340/Tuf890 were used for the first PCR and Tuf400/Tuf835 for the nested PCR (**Table 1**). M13 and T7 sequences were attached to the inner primer pair to facilitate sequencing. The nested PCR resulted in products of the expected size (420–444 bp) from all 91 phytoplasma strains tested (**Table S1)** and no products from a range of healthy plant controls (**Figure 1**). The *tuf* gene PCR products were sequenced and the obtained sequences were deposited in the NCBI GenBank and in the QBOL project reference barcode database Q-bank (www.q-bank.eu).

### Sequence Analysis

The sequences of the *tuf* barcode and, for comparison, the 1,240 bp R16F2n/R16R2 fragment of the 16S rRNA gene were assembled into two datasets for sequence analysis. The *tuf* barcode dataset consisted of sequences from 91 phytoplasma strains obtained in this study, whereas the 16S rRNA gene dataset contained sequences from 66 selected strains that were sequenced in this study or imported from NCBI Genbank. Both datasets included sequences from 16Sr groups -I, -II, -III, -IV, -V, -VI, -VII, -IX, -X, -XI, -XII, -XV, -XX.

**Figure 3:**
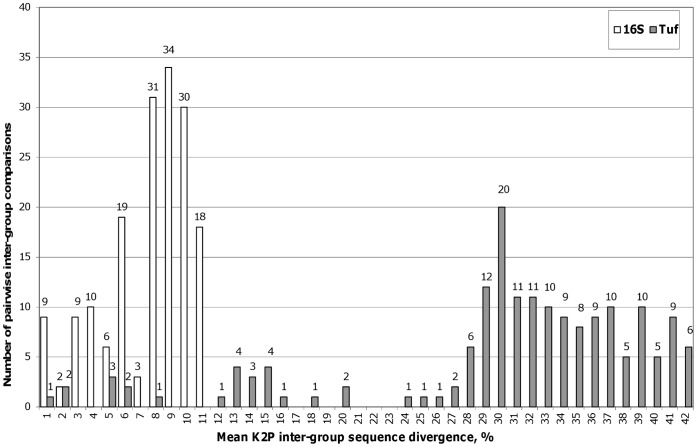
Distribution of the pairwise inter-group mean K2P sequence divergence. Pairwise Kimura-2-parameter average distances between groups were determined for 91 and 66 phytoplasma strains from 19 groups for *tuf* and 16Sr, respectively. Note that inter-group sequence divergence in the 420–444 bp *tuf* barcode is much higher than divergence in the 1,2 kbp 16Sr gene fragment.

Average inter-group K2P evolutionary divergence was calculated for nineteen phytoplasma groups (16Sr groups or ‘*Candidatus*’ species). Distribution of mean inter-group sequence divergence revealed that most of the 16Sr pairwise comparisons had 3–11% sequence divergence, whereas for *tuf* they ranged 28–42%, suggesting that the *tuf* barcode has more characters that allow better separation among groups (**Figure 3** **)**. However, in several instances, pairwise inter-group comparisons were as low as 1–8% for the *tuf* barcode. These outliers represent phytoplasma groups, which are closely related to each other, but are considered separate ‘*Candidatus’* species based on distinctive biological and phytopathological properties [3], and include phytoplasmas from the 16SrV (A, B, C+D, E) and 16SrX (A, B, C) groups. Taken together, these results suggest that although the *tuf* barcode demonstrates a variable level of ribosomal subgroup resolution, its overall performance is comparable with the full 16Sr sequence or better.

**Figure 4:**
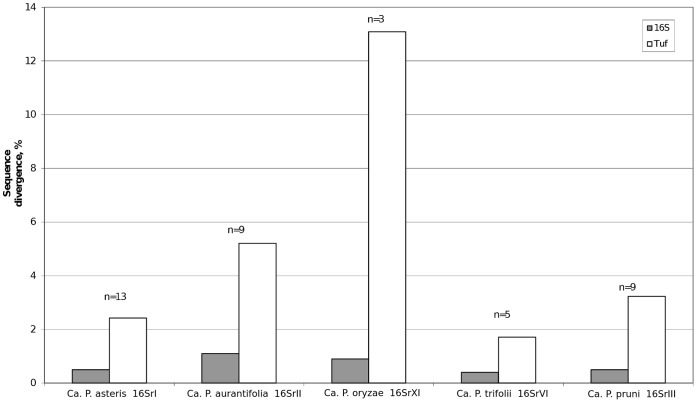
Average K2P within-group sequence divergence. ‘*Ca.* P. oryzae’ and ‘*Ca.* P. aurantifolia’ showed considerably higher intra-group divergences, suggesting the presence of subgroups in these groups, not recognized by the 16Sr-based phylogeny. Average Kimura-2-parameter distances were calculated for the 16S rRNA and *tuf* sequences of the same strains, n – number of phytoplasma strains within a group. Strains used in the analysis: ‘*Ca.* P. asteris’ group 16SrI – A-YA, CA, KVE, CHRYM, HYDP, NJ-AY, GD-1, AY-1, AYBG, RV, AY-J24126, AY-2192, AVUT; ‘*Ca.* P. aurantifolia’ group 16SrII – PEP, SPLL, SEPT, TBB-KG, WBDL, CoP, PrB, FAP, VCP; ‘*Ca.* P. oryzae’ group 16SrXI – NGS, NGS-BS, BVK; ‘*Ca*. P. trifolii’ group 16SrVI – CP-1, PWB, LUM, BLL, CPS; ‘*Ca*. P. pruni’ group 16SrIII – MW1, VAC, GR1, LNI, CR, SP1, CX, BF, GVX.

Average within-group sequence divergence was determined for groups where *tuf* and 16Sr sequences were available for at least three strains (16SrI, -II, -III, -VI and -XI) (**Figure 4**). The highest variation was found in the *tuf* dataset groups ‘*Ca.* P. oryzae’ group 16SrXI (13.1%) and ‘*Ca.* P. aurantifolia’ group 16SrII (4.9%), suggesting the presence of phytoplasma subgroups which were not identified based on 16S rRNA gene phylogeny alone, which was also observed in other studies of group 16SrII [19], [43]. However, we cannot rule out the possibility that the combination of highly variable sequences and a low number of representatives in a given group could artificially inflate average intra-group sequence divergence. Comparison of the *tuf* barcode average inter- (both minimal and mean values) and intra- group K2P sequence divergence revealed the presence of a barcoding gap in most groups, as ratios between inter- and intra-group divergences were greater than 1 (**Table 2**).

**Table 2:** Comparison of the *tuf* barcode mean K2P intra- and inter-group divergences.

Phytoplasma group	Number of strains	K2P mean inter-group distance		K2P intra-group distance	Average inter−/intra-d ratio
		min	average		
‘Ca. P. asteris’ 16SrI	16	0.129	0.322	0.024	13.418
‘Ca. P. aurantifolia’ 16SrII	10	0.128	0.380	0.049	7.753
‘Ca. P. pruni’ 16SrIII	11	0.272	0.315	0.028	11.247
Coconut lethal yellows 16SrIV	2	0.239	0.349	0.003	116.236
‘Ca. P. ulmi’ 16SrV-A	3	0.021	0.264	0	n/a
‘Ca. P. ziziphi’ 16SrV-B	1	0.061	0.265	n/c	n/c
‘Flavescence dorée’ 16SrV-C and -D	13	0.005	0.253	0.003	84.499
‘Ca. P.rubi’ 16SrV-E	1	0.005	0.253	n/c	n/c
‘Ca. P. trifolii’ 16SrVI	5	0.116	0.263	0.016	16.453
‘Ca. P. fraxini’ 16SrVII	4	0.116	0.256	0.020	12.821
‘Ca. P. phoenicium’ 16SrIX	2	0.257	0.332	0	n/a
‘Ca. P. mali’ 16SrX-A	3	0.049	0.295	0	n/a
‘Ca. P. prunorum’ 16SrX-B	2	0.050	0.301	0	n/a
‘Ca. P. pyri’ 16SrX-C	2	0.049	0.288	0	n/a
‘Ca. P. oryzae’ 16SrXI	3	0.285	0.352	0.131	2.687
Stolbur 16SrXII	10	0.134	0.313	0	n/a
‘Ca. P. australiense’ 16SrXII-B	1	0.129	0.332	n/c	n/c
‘Ca. P. brasiliense’ 16SrXV	1	0.128	0.364	n/c	n/c
‘Ca. P. rhamni’ 16SrXX	1	0.181	0.312	n/c	n/c

K2P genetic distances were calculated within (intra) and between (inter) phytoplasma groups. Minimum and average mean K2P inter-group divergences were calculated for all phytoplasma groups. Intra-group divergences could only be calculated for groups with more than one representative. K2P inter-group distance values greater than K2P intra-group distance values (or average inter−/intra-group divergence ratios >1) indicate that inter- and intra- group divergences do not overlap and suggest the presence of a barcoding gap. d, sequence divergence distance; n/a, not available; n/c, not calculated.

The obtained *tuf* sequences were subjected to phylogenetic analysis to further test whether individual sequences form groups that can be used for identification. However, it should be stressed that the *tuf* sequences used in this study were solely intended for identification of phytoplasmas and not for phylogenetics. Neighbor-Joining trees constructed from the *tuf* and 16S rRNA alignments showed remarkable similarity in terminal taxa, implying that the *tuf* barcode is well linked to the existing 16S rRNA phytoplasma phylogeny (**Figure 2**). This was supported by a separate phylogenetic analysis using the Maximum Likelihood method (**Figure S1 and Figure S2**).

Furthermore, the *tuf* -based NJ phylogeny also resolved subgroups within several 16Sr groups. For example, 16SrII could be split into several subgroups (**Figure 2** **A**), as reported previously [19], [43]. Another example is the 16SrX group that was resolved into the three ‘*Candidatus*’ species: ‘*Ca.* P. pyri’, ‘*Ca.* P. mali’ and ‘*Ca.* P. prunorum’, all of which are important pathogens of fruit trees in Europe. ‘*Ca.* P. asteris’ (16SrI), which has previously been divided into subgroups based on the RFLP analysis of the 16S rRNA region, could clearly be differentiated into subgroups 16SrI-A, -B and -C using the *tuf* barcode. Closely related 16SrV group members containing important pathogens such as ‘flavescence doreé’ (16SrV-C and -D) and elm yellows (16SrV-A) could also be separated using the *tuf* barcode (**Figure 2** **A)**, as seven single nucleotide polymorphisms (SNPs) were observed between ‘flavescence doreé’ and elm yellows and one SNP was found between subgroups 16SrV-C/D and 16SrV-E (‘*Ca*. P. rubi’).

In conclusion, it was demonstrated that the *tuf* barcode, being three times shorter than the full length 16Sr gene, has much higher both inter- and intra- group divergence than the 16Sr gene and that inter- and intra- group divergences did not overlap creating a barcoding gap, one of the prerequisites for newly proposed DNA barcodes [44]. Furthermore, phylogenetic analysis and alignments showed that important groups of phytoplasmas could readily be identified. All these findings suggest that while being shorter, the *tuf* barcode provides clear resolution at both group and subgroup levels compared to the 16S rRNA gene. Finally, it should be noted that in the case of mixed infection, it may not be possible to obtain good quality sequence information, however, this is a general problem in phytoplasma research that may be solved by cloning of PCR products and subsequent sequencing of individual clones. The sensitivity of the PCR using *tuf* primers was not tested in this study, however, the use of a small fragment likely increases sensitivity in PCR compared to much larger fragments used previously in other phytoplasma studies.

### Online Identification Tool and DNA Barcode Database

This work was a part of the QBOL initiative, which aims to adopt DNA barcoding principles to identification of plant pests and pathogens with a focus on quarantine organisms and to develop a DNA barcode-based identification system [27]. This includes establishment of a free online reference sequence database. The DNA barcodes obtained in this study were deposited in the database of plant pests and pathogens and can be found on http://www.q-bank.eu/Phytoplasmas/together with other relevant information (geographical origin of strains, original and maintenance hosts, 16Sr groups and subgroups etc). This DNA barcoding procedure will provide plant inspection services and other diagnosticians with a robust and easily performed identification tool. By using the protocols and primers provided on the mentioned above website, it will be possible to sequence phytoplasma DNA and with the help of the online identification tool to compare sequences from field-collected phytoplasmas with reference strain sequences. This DNA barcoding system will improve detection of phytoplasmas associated with plant diseases.

## Supporting Information

Figure S1 **The bootstrap Maximum Likelihood tree of the** ***tuf*** **barcode.** The ML tree supports the terminal branches clustering according to the 16Sr phytoplasma group classification observed in the NJ tree (**Figure 2a**). The tree is drawn to scale, with branch lengths measured in the number of substitutions per site. Numbers at the nodes indicate bootstrap values; bar, substitutions per nucleotide position; 16Sr group and subgroup are in parentheses; *A. laidlawii* (accession number NC010163) was used as an outgroup.
(PDF)

Figure S2 **The bootstrap Maximum Likelihood tree of the R16F2n/R16R2 fragment of the 16S ribosomal RNA gene.** The ML tree supports the terminal branches clustering according to the 16Sr phytoplasma group classification observed in the NJ tree (**Figure 2b**). The tree is drawn to scale, with branch lengths measured in the number of substitutions per site. Numbers at the nodes indicate bootstrap values; bar, substitutions per nucleotide position; asterisk, strains sequenced in this study; GenBank sequence accession number is indicated following the strain acronym; 16Sr group and subgroup are in parentheses; *A. laidlawii* (accession number NC010163) was used as an outgroup.
(PDF)

Table S1 Phytoplasma strains used in this study.
(XLS)

Table S2 Maximum Likelihood fits of 24 different nucleotide substitution models calculated for the *tuf* barcode. Models with the lowest BIC scores (Bayesian Information Criterion) are considered to describe the substitution pattern the best. Abbreviations: GTR, General Time Reversible; HKY, Hasegawa-Kishino-Yano; TN93, Tamura-Nei; T92, Tamura 3-parameter; K2, Kimura 2-parameter; JC, Jukes-Cantor; #Param, number of parameters; AICc, Akaike Information Criterion, corrected; lnL, Maximum Likelihood value; +G, estimates of gamma shape parameter 5 rate categories; +I, an estimated fraction of invariant sites; R, assumed or estimated values of transition/transversion bias; f, nucleotide frequencies; r, rates of base substitutions for each nucleotide pair; n/a, not available.
(XLS)

Table S3 Maximum Likelihood fits of 24 different nucleotide substitution models calculated for the R16F2n/R16R2 fragment of the 16S ribosomal RNA gene. Models with the lowest BIC scores (Bayesian Information Criterion) are considered to describe the substitution pattern the best. Abbreviations: GTR, General Time Reversible; HKY, Hasegawa-Kishino-Yano; TN93, Tamura-Nei; T92, Tamura 3-parameter; K2, Kimura 2-parameter; JC, Jukes-Cantor; #Param, number of parameters; AICc, Akaike Information Criterion, corrected; lnL, Maximum Likelihood value; +G, estimates of gamma shape parameter 5 rate categories; +I, an estimated fraction of invariant sites; R, assumed or estimated values of transition/transversion bias; f, nucleotide frequencies; r, rates of base substitutions for each nucleotide pair; n/a, not available.
(XLS)

## References

[1] Bertaccini A (2007) Phytoplasmas: diversity, taxonomy, and epidemiology. Front Biosci 12: 673–689. 17127328 10.2741/2092

[2] Hogenhout SA, Oshima K, Ammar ED, Kakizawa S, Kingdom H, et al (2008) Phytoplasmas: bacteria that manipulate plants and insects. Mol Plant Pathol 9: 403–423. 18705857 10.1111/j.1364-3703.2008.00472.x PMC6640453

[3] IRPCM Phytoplasma/Spiroplasma Working Team – Phytoplasma taxonomy group (2004) ‘*Candidatus* Phytoplasma’, a taxon for the wall-less, non-helical prokaryotes that colonize plant phloem and insects. Int J Syst Evol Microbiol 54: 1243–1255. 15280299 10.1099/ijs.0.02854-0

[4] Wei W, Davis RE, Lee I-M, Zhao Y (2007) Computer-simulated RFLP analysis of 16S rRNA genes: identification of ten new phytoplasma groups. Int J Syst Evol Microbiol 57: 1855–1867. 17684271 10.1099/ijs.0.65000-0

[5] Lee I-M, Gundersen-Rindal DE, Davis RE, Bartoszyk IM (1998) Revised classification scheme of phytoplasmas based on RFLP analyses of 16S rRNA and ribosomal protein gene sequences. Int J Syst Bacteriol 48: 1153–1169. 10.1099/00207713-48-1-269 9542097

[6] Gundersen DE, Lee I-M, Schaff DA, Harrison NA, Chang CJ, et al (1996) Genomic diversity and differentiation among phytoplasma strains in 16S rRNA groups I (aster yellows and related phytoplasmas) and III (X-disease and related phytoplasmas). Int J Syst Bacteriol 46: 64–75. 8573523 10.1099/00207713-46-1-64

[7] Deng S, Hiruki C (1991) Amplification of 16S rRNA genes from culturable and nonculturable mollicutes. J Microbiol Methods 14: 53–61.

[8] Schneider B, Seemüller E, Smart CD, Kirkpatrick BC (1995) Phylogenetic classification of plant pathogenic mycoplasma-like organisms or phytoplasmas. In: Razin S, Tully JG, editors. Molecular and diagnostic procedures in mycoplasmology. San Diego, CA: Academic press. 369–380.

[9] Liefting LW, Andersen MT, Beever RE, Gardner RC, Forster RL (1996) Sequence heterogeneity in the two 16S rRNA genes of *Phormium* yellow leaf phytoplasma. Appl Environ Microbiol 62: 3133–3139. 8795200 10.1128/aem.62.9.3133-3139.1996 PMC168106

[10] Marcone C, Lee I-M, Davis RE, Ragozzino A, Seemüller E (2000) Classification of aster yellows-group phytoplasmas based on combined analyses of rRNA and *tuf* gene sequences. Int J Syst Evol Microbiol 50: 1703–1713. 11034478 10.1099/00207713-50-5-1703

[11] Botti S, Bertaccini A (2003) Variability and functional role of chromosomal sequences in 16SrI-B subgroup phytoplasmas including aster yellows and related strains. J Appl Microbiol 94: 103–110. 12492930 10.1046/j.1365-2672.2003.01809.x

[12] Lee I-M, Zhao Y, Bottner KD (2006) *SecY* gene sequence analysis for finer differentiation of diverse strains in the aster yellows phytoplasma group. Mol Cell Probes 20: 87–91. 16330183 10.1016/j.mcp.2005.10.001

[13] Mitrović J, Contaldo N, Paltrinieri S, Meja JF, Mori N, et al (2011) The use of *groEL* gene in characterisation of aster yellows phytoplasmas in field collected samples. Bull Insectology 64: S17–S18.

[14] Arnaud G, Malembic-Maher S, Salar P, Bonnet P, Maixner M, et al (2007) Multilocus sequence typing confirms the close genetic interrelatedness of three distinct flavescence dorée phytoplasma strain clusters and group 16SrV phytoplasmas infecting grapevine and alder in Europe. Appl Environ Microbiol 73: 4001–4010. 17468266 10.1128/AEM.02323-06 PMC1932733

[15] Martini A, Botti S, Marcone C, Marzachì C, Casati P, et al (2002) Genetic variability among Flavescence dorée phytoplasmas from different origins in Italy and France. Mol Cell Probes 16: 197–208. 12144771 10.1006/mcpr.2002.0410

[16] Streten C, Gibb KS (2005) Genetic variation in ‘*Candidatus* Phytoplasma australiense’. Plant Pathol 54: 8–14.

[17] Martini M, Lee I-M, Bottner KD, Zhao Y, Botti S, et al (2007) Ribosomal protein gene-based phylogeny for finer differentiation and classification of phytoplasmas. Int J Syst Evol Microbiol 57: 2037–2051. 17766869 10.1099/ijs.0.65013-0

[18] Lee I-M, Bottner-Parker KD, Zhao Y, Davis RE, Harrison NA (2010) Phylogenetic analysis and delineation of phytoplasmas based on *secY* gene sequences. Int J Syst Evol Microbiol 60: 2887–2897. 20097798 10.1099/ijs.0.019695-0

[19] Hodgetts J, Boonham N, Mumford R, Harrison N, Dickinson M (2008) Phytoplasma phylogenetics based on analysis of *secA* and 23S rRNA gene sequences for improved resolution of candidate species of ‘*Candidatus* Phytoplasma’. Int J Syst Evol Microbiol 58: 1826–1837. 18676464 10.1099/ijs.0.65668-0

[20] Hebert PDN, Cywinska A, Ball SL, deWaard JR (2003) Biological identifications through DNA barcodes. Proc Biol Sci 270: 313–321. 12614582 10.1098/rspb.2002.2218 PMC1691236

[21] Besansky NJ, Severson DW, Ferdig MT (2003) DNA barcoding of parasites and invertebrate disease vectors: what you don’t know can hurt you. Trends Parasitol 19: 545–546. 14642760 10.1016/j.pt.2003.09.015

[22] Casiraghi M, Labra M, Ferri E, Galimberti A, De Mattia F (2010) DNA barcoding: a six-question tour to improve users’ awareness about the method. Brief Bioinform 11: 440–453. 20156987 10.1093/bib/bbq003

[23] Hebert PDN, Ratnasingham S, deWaard JR (2003) Barcoding animal life: cytochrome *c* oxidase subunit 1 divergences among closely related species. Proc Biol Sci 270 Suppl: S96–S99 10.1098/rsbl.2003.0025 PMC1698023 12952648

[24] Hebert PDN, Penton EH, Burns JM, Janzen DH, Hallwachs W (2004) Ten species in one: DNA barcoding reveals cryptic species in the neotropical skipper butterfly *Astraptes fulgerator* . Proc Natl Acad Sci U S A 101: 14812–14817. 15465915 10.1073/pnas.0406166101 PMC522015

[25] Kress WJ, Wurdack KJ, Zimmer EA, Weigt LA, Janzen DH (2005) Use of DNA barcodes to identify flowering plants. Proc Natl Acad Sci U S A 102: 8369–8374. 15928076 10.1073/pnas.0503123102 PMC1142120

[26] Vernooy R, Haribabu E, Muller MR, Vogel JH, Hebert PDN, et al (2010) Barcoding life to conserve biological diversity: beyond the taxonomic imperative. PLoS Biol 8: e1000417. 20644709 10.1371/journal.pbio.1000417 PMC2903590

[27] Bonants P, Groenewald E, Rasplus JY, Maes M, de Vos P, et al (2010) QBOL: a new EU project focusing on DNA barcoding of quarantine organisms. EPPO Bulletin 40: 30–33.

[28] Oshima K, Kakizawa S, Nishigawa H, Jung HY, Wei W, et al (2004) Reductive evolution suggested from the complete genome sequence of a plant-pathogenic phytoplasma. Nat Genet 36: 27–29. 14661021 10.1038/ng1277

[29] Bai X, Zhang J, Ewing A, Miller SA, Radek AJ, et al (2006) Living with genome instability: the adaptation of phytoplasmas to diverse environments of their insect and plant hosts. J Bacteriol 188: 3682–3696. 16672622 10.1128/JB.188.10.3682-3696.2006 PMC1482866

[30] Tran-Nguyen LTT, Kube M, Schneider B, Reinhardt R, Gibb KS (2008) Comparative genome analysis of ‘*Candidatus* Phytoplasma australiense’ (subgroup tuf-Australia I; rp-A) and ‘*Ca*. Phytoplasma asteris’ strains OY-M and AY-WB. J Bacteriol 190: 3979–3991. 18359806 10.1128/JB.01301-07 PMC2395047

[31] Kube M, Schneider B, Kuhl H, Dandekar T, Heitmann K, et al (2008) The linear chromosome of the plant-pathogenic mycoplasma ‘*Candidatus* Phytoplasma mali’. BMC Genomics 9: 306. 18582369 10.1186/1471-2164-9-306 PMC2459194

[32] Bertaccini A (2010) Phytoplasma Collection. Available: http://ipwgnet.org/doc/phyto_collection/collection-august2010.pdf. Accessed 24 February 2012.

[33] Prince JP, Davis RE, Wolf TK, Lee I-M, Mogen BD, et al (1993) Molecular detection of diverse mycoplasmalike organisms (MLOs) associated with grapevine yellows and their classification with Aster Yellows, X-disease, and Elm Yellows MLOs. Phytopathology 83: 1130–1137.

[34] Gibb KS, Padovan AC, Mogen BD (1995) Studies on sweet potato little-leaf phytoplasma detected in sweet potato and other plant species growing in Northern Australia. Mol Plant Pathol 85: 169–174.

[35] Gundersen DE, Lee I-M (1996) Ultrasensitive detection of phytoplasmas by nested-PCR assays using two universal primer pairs. Phytopathol Mediterr 35: 144–151.

[36] Feng DF, Doolittle RF (1987) Progressive sequence alignment as a prerequisite to correct phylogenetic trees. J Mol Evol 25: 351–360. 3118049 10.1007/BF02603120

[37] Tamura K, Dudley J, Nei M, Kumar S (2007) MEGA4: Molecular Evolutionary Genetics Analysis (MEGA) software version 4.0. Mol Biol Evol 24: 1596–1599. 17488738 10.1093/molbev/msm092

[38] Saitou N, Nei M (1987) The neighbor-joining method: a new method for reconstructing phylogenetic trees. Mol Biol Evol 4: 406–425. 3447015 10.1093/oxfordjournals.molbev.a040454

[39] Felsenstein J (1985) Confidence limits on phylogenies: An approach using the bootstrap. Evolution 39: 783–791. 28561359 10.1111/j.1558-5646.1985.tb00420.x

[40] Kimura M (1980) A simple method for estimating evolutionary rates of base substitutions through comparative studies of nucleotide sequences. J Mol Evol 16: 111–120. 7463489 10.1007/BF01731581

[41] Tamura K, Nei M (1993) Estimation of the number of nucleotide substitutions in the control region of mitochondrial DNA in humans and chimpanzees. Mol Biol Evol 10: 512–526. 8336541 10.1093/oxfordjournals.molbev.a040023

[42] Tamura K (1992) Estimation of the number of nucleotide substitutions when there are strong transition-transversion and G+C-content biases. Mol Biol Evol 9: 678–687. 1630306 10.1093/oxfordjournals.molbev.a040752

[43] Khan AJ, Botti S, Al-Subhi AM, Gundersen-Rindal DE, Bertaccini AF (2002) Molecular identification of a new phytoplasma associated with alfalfa witches’-broom in Oman. Phytopathology 92: 1038–1047. 18944213 10.1094/PHYTO.2002.92.10.1038

[44] Hebert PDN, Stoeckle MY, Zemlak TS, Francis CM (2004) Identification of birds through DNA barcodes. PLoS Biol 2: e312. 15455034 10.1371/journal.pbio.0020312 PMC518999

