# Supplementary figures and images for: DNA Barcoding for Identification of ‘Candidatus Phytoplasmas’ Using a Fragment of the Elongation Factor Tu Gene
Source: PLoS One. 2012 Dec 18;7(12):e52092. doi: 10.1371/journal.pone.0052092 (PMC3525539; doi:10.1371/journal.pone.0052092)

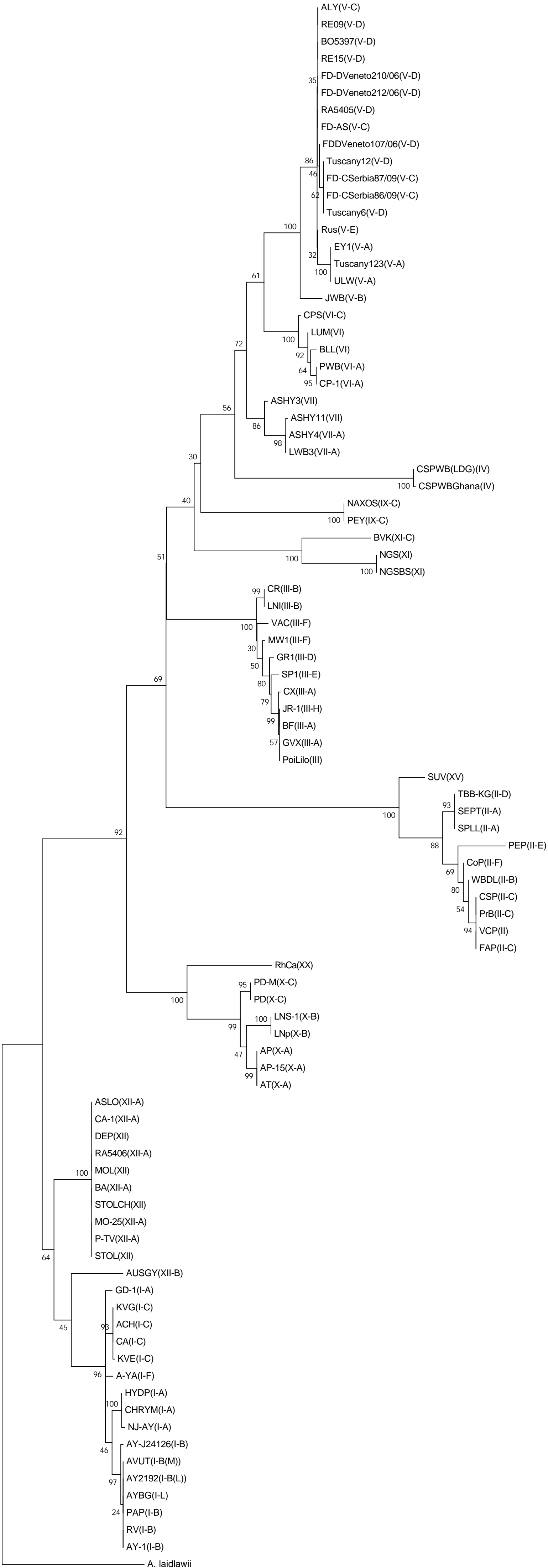

Supplement: Figure S1 — The bootstrap Maximum Likelihood tree of the tuf barcode. The ML tree supports the terminal branches clustering according to the 16Sr phytoplasma group classification observed in the NJ tree ( Figure 2a ). The tree is drawn to scale, with branch lengths measured in the number of substitutions per site. Numbers at the nodes indicate bootstrap values; bar, substitutions per nucleotide position; 16Sr group and subgroup are in parentheses; A. laidlawii (accession number NC010163) was used as an outgroup. (PDF) [file pone.0052092.s001.pdf]

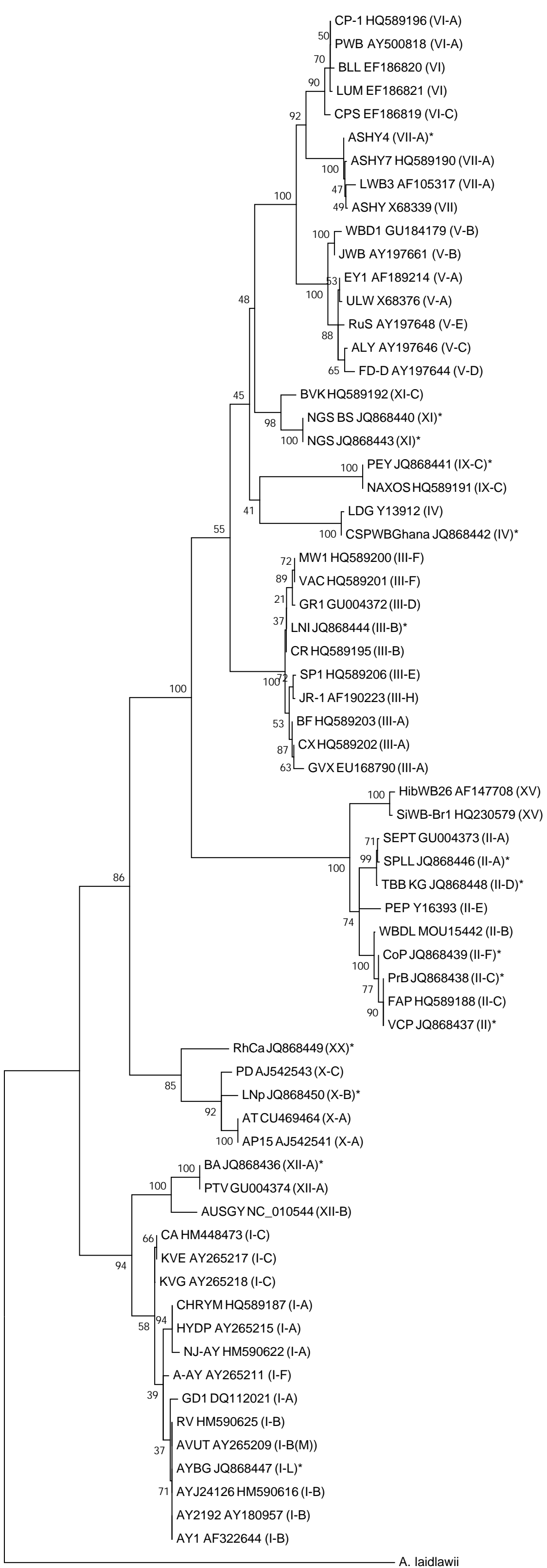

Supplement: Figure S2 — The bootstrap Maximum Likelihood tree of the R16F2n/R16R2 fragment of the 16S ribosomal RNA gene. The ML tree supports the terminal branches clustering according to the 16Sr phytoplasma group classification observed in the NJ tree ( Figure 2b ). The tree is drawn to scale, with branch lengths measured in the number of substitutions per site. Numbers at the nodes indicate bootstrap values; bar, substitutions per nucleotide position; asterisk, strains sequenced in this study; GenBank sequence accession number is indicated following the strain acronym; 16Sr group and subgroup are in parentheses; A. laidlawii (accession number NC010163) was used as an outgroup. (PDF) [file pone.0052092.s002.pdf]
